# Synergistic effects of *Ferula asafoetida* extract and condensed tannins from raisin pomace on *in vitro* cecal fermentation kinetics and nutrient digestibility in horses

**DOI:** 10.14202/vetworld.2026.905-919

**Published:** 2026-03-13

**Authors:** Hossein Dehghan, Sima Moghaddaszadeh-Ahrabi, Hossein Hashemzadeh-Farhang, Parisa Shahbazi, Babak Nobari

**Affiliations:** 1 Department of Animal Science, Ta.C., Islamic Azad University, Tabriz, Iran; 2 Department of Pathobiology, TaMS.C., Islamic Azad University, Tabriz, Iran; 3 Department of Pathobiology, Faculty of Veterinary Medicine, University of Tabriz, Tabriz, Iran; 4 Registered Animal Nutritionist at Nutrition Society of Australia, Melbourne, Victoria, Australia

**Keywords:** acidosis, cecal fermentation, condensed tannins, equine nutrition, *Ferula asafoetida*, horse feed additives, *in vitro* gas production, nutrient digestibility, raisin pomace tannins

## Abstract

**Background and Aim:**

The equine hindgut depends on microbial fermentation for efficient nutrient utilization but remains vulnerable to dysbiosis, hindgut acidosis, and suboptimal fiber digestion. Growing restrictions on antibiotic and synthetic feed additives have increased interest in natural phytogenic compounds. Medicinal plant extracts and condensed tannins are promising candidates to modulate microbial activity, improve fermentation efficiency, and enhance nutrient digestibility. This study aimed to investigate the individual and combined effects of hydroalcoholic extract of *Ferula asafoetida* and condensed tannins extracted from raisin pomace on equine cecal fermentation parameters and nutrient utilization using *in vitro* gas production and batch culture techniques.

**Materials and Methods:**

A 2 × 2 factorial *in vitro* design was used with four treatments: control (C; basal diet only), *F. asafoetida* extract (A; 30 mg), condensed tannins from raisin pomace (G; 50 mg), and their combination (A × G). Fecal inoculum was collected from four healthy 14-month-old Arabian geldings adapted for 14 days to a forage-based maintenance diet. Fermentation kinetics were evaluated over 120 h using the *in vitro* gas production technique and fitted to the Gompertz model. Parallel batch cultures measured pH, ammonia-nitrogen (NH_3_-N), and apparent disappearances of dry matter (DM), crude protein (CP), acid detergent fiber (ADF), and neutral detergent fiber (NDF). Data were analyzed using PROC GLM in SAS with Tukey–Kramer post-hoc tests (p < 0.05).

**Results:**

Cumulative gas production at 120 h was significantly higher in G (340.5 mL) and A × G (340.3 mL) than in C (228.8 mL) (p < 0.01), with faster fermentation rates and shorter lag times (p < 0.01). Terminal pH values remained stable (6.33–6.40) across treatments with no indication of acidosis. NH_3_-N concentrations were elevated in G (26.0 mg/dL) and A × G (25.5 mg/dL) compared with C (24.5 mg/dL) (p < 0.01). Apparent digestibility improved markedly: DM increased from 64.5% (C) to 70.3% (G), CP from 60.3% (C) to 66.9% (G), with parallel positive trends observed for ADF and NDF (p < 0.01).

**Conclusion:**

Supplementation with *F. asafoetida* extract and condensed tannins from raisin pomace, especially in combination, enhanced fermentation efficiency, accelerated substrate degradation, and improved nutrient digestibility while maintaining stable pH in an *in vitro* equine cecal model. These findings indicate strong potential for these phytogenic compounds as sustainable natural feed additives to optimize equine hindgut function. *In vivo* validation, dose optimization, and long-term microbiome studies are recommended to confirm practical efficacy and safety in horses.

## INTRODUCTION

Horses have long been integral to human society as companions, athletes, and contributors to agriculture and transport. Ensuring their health and performance is essential, with gastrointestinal function playing a central role due to their dependence on hindgut microbial fermentation for nutrient absorption. However, the complexity of the equine digestive system makes it susceptible to disorders such as colic, diarrhea, and parasitic infections, which can limit productivity and lifespan [1, 2].

Amid growing restrictions on synthetic growth promoters and the global rise of antimicrobial resistance, the equine industry is urgently seeking natural alternatives [3, 4]. This study proposes a novel phytobiotic–phytonutrient synergy by combining *Ferula asafoetida* extract and condensed tannins derived from grape (raisin) pomace. This approach aims to modulate hindgut fermentation through natural mechanisms, offering a sustainable solution for the antibiotic-free nutrition movement [3, 4]. Furthermore, valorizing agro-industrial by-products is critical for sustainable animal nutrition. In Iran, particularly in Yazd Province, the raisin processing industry generates substantial quantities of pomace, a residue often discarded despite its high phenolic content [5]. Repurposing this local by-product transforms an environmental burden into a cost-effective, bioactive feed additive, aligning with circular economy principles to support the equine industry.

To the best of our knowledge, this is the first study to investigate the specific effects of *F. asafoetida* and raisin pomace tannins on equine cecal fermentation kinetics using a controlled *in vitro* system. Despite the established benefits of these plants in ruminants, a critical knowledge gap exists regarding their application in equine nutrition. The combined effect of *F. asafoetida* and raisin pomace tannins on the distinct fermentation physiology of the equine cecum remains uncharacterized.

To address this, we evaluated the synergistic potential of these extracts using *in vitro* gas production and batch culture techniques. We hypothesized that the bioactive compounds in *F. asafoetida* and the protein-binding tannins in grape pomace would act synergistically to optimize fermentation kinetics and enhance nutrient digestibility while maintaining a stable cecal pH. Accordingly, we determined the effects of this phytogenic blend on gas production profiles, ammonia-nitrogen concentrations, and the apparent digestibility of fiber and protein.

## MATERIALS AND METHODS

### Ethical approval

All experimental procedures, specifically animal handling and biological sampling, were approved by the Animal Ethics Committee at the Islamic Azad University of Tabriz (approval number 1401/A/IUAT/P134). The study design was in accordance with the national regulations and ethical guidelines of the Iranian Institute of Animal Science.

All participating researchers completed certified training in animal welfare and laboratory biosafety prior to field work. The Department of Animal Science at Islamic Azad University of Tabriz (IAUT) supervised the operations to ensure strict adherence to the 3Rs and proper biological waste disposal protocols. The owner of the private stud farm in Yazd Province provided written consent for the collection of samples from the Arabian foals (Contract No. 210/14BU/14010123).

### Study period and location

Field sampling was conducted in spring 2020 at a private Arabian horse breeding farm located in the Ashk-e-Zar district of Yazd Province, Iran. Subsequent laboratory analyses were performed at Yazd Mokamel Co.’s advanced animal nutrition laboratory, Yazd, Iran.

### Experimental design

This study employed a 2 × 2 factorial *in vitro* design using fecal inoculum obtained from four Arabian foals. The experimental workflow integrated the hydroalcoholic extraction of *F. asafoetida* and aqueous acetone extraction of raisin pomace tannin solution (RPS) with parallel fermentation assays. *In vitro* gas production and *in vitro* batch culture techniques were used to assess fermentation kinetics, pH stability, NH_3_-N, and nutrient digestibility, with a general linear model being used to analyze all parameters.

This study investigated the effects of the alcoholic extract of Asafoetida (*F. asafoetida* L.) and tannins derived from raisin pomace on the fermentation pattern and digestive responses in the routine diets of horses. The experiments included the following:

1. *In vitro* gas production: fermentation characteristics of experimental diets were measured.
2. *In vitro* batch culture analysis: fermentation profile, pH, and NH_3_-N changes were evaluated.

### Asafoetida extraction from the *F. asafoetida* plant

Specimens of *F. asafoetida* L. (Apiaceae) were harvested during the spring of 2020 from natural habitats in the Yazd and Kerman provinces, Iran. A taxonomist at the Medicinal Plant Research Institute authenticated the plant material, and a voucher specimen was deposited at the herbarium for future reference.

Extraction was performed using a modified hydroalcoholic maceration technique based on standard phytochemical protocols [6]. Briefly, the collected plant material was shade-dried for 10 days to minimize the thermal degradation of volatile compounds. The dried material was milled using a hammer mill (Retsch HM 200, Haan, Germany) and passed through a 2-mm mesh sieve. For extraction, 100 g of the powdered material was macerated in 70% ethanol (v/v) at a solid-to-solvent ratio of 1:10. The suspension was maintained at room temperature (25°C) for 7 days, with manual stirring performed twice daily to facilitate mass transfer. To ensure exhaustive extraction, the solvent was renewed every 48 h (the process was repeated four times). The combined supernatants were filtered (Whatman No. 1) and concentrated under reduced pressure using a rotary evaporator (Büchi Rotavapor® R-80, Flawil, Switzerland) at 45°C–50°C. The overall extraction yield was approximately 15% (w/w, dry matter (DM) basis), expressed as the crude extract per 100 g of dried plant material. The resulting dark-brown, semi-solid OG-resin extract was stored in airtight, amber glass containers at 4°C until analysis.

The extraction protocols were designed to isolate soluble bioactive metabolites, thereby removing structural carbohydrates. Consequently, the extracts were considered devoid of significant neutral detergent fiber (NDF)/acid detergent fiber (ADF), and their inclusion at milligram levels had a negligible impact on the gross macronutrient composition of the basal diet.

### Gas chromatography coupled with mass spectrometry (GC-MS) analysis of *F. asafoetida* active compounds

The chemical composition of the *F. asafoetida* extract was determined using GC-MS. Analysis was conducted at the Medicinal Plant Research Institute (Karaj, Iran) using a Thermo Fisher Scientific Trace GC Ultra system coupled with an ISQ Single Quadrupole Mass Spectrometer. Prior to analysis, a 100 mg aliquot of the freshly prepared extract was diluted to 1 mL with analytical-grade methanol and filtered through a 0.22 µm PTFE syringe filter. A 1.0 µL volume was injected in split mode (split ratio 1:10) with an injector temperature of 250°C. Separation was achieved on an HP-5MS capillary column (30 m x 0.25 mm i.d., 0.25 µm film thickness; Agilent Technologies, USA). The oven temperature program was initiated at 60°C (held for 3 min), then increased to 240°C at 4°C/min and held at 240°C for 10 min. Helium (99.999%) was used as the carrier gas at a constant flow rate of 1.0 mL/min. Mass spectra were acquired in electron impact mode at an ionization energy of 70 eV, a scan range of 40–500 m/z and an ion source temperature of 230°C. Compounds were identified by comparing their mass spectral fragmentation patterns and Kovats retention indices (RI) with those stored in the National Institute of Standards and Technology (NIST) and Wiley mass spectral libraries [7].

### Extraction and quantification of tannin extracts

In the spring of 2020, fresh raisin pomace (*Vitis vinifera* L.) was sourced from a mixed dried fruit and nut processing facility in Yazd, Iran. To minimize tannin oxidation, samples were collected immediately after processing and stored at 4°C, with bioactive retention subsequently confirmed via tannin analysis.

The collected pomace was sun-dried for seven days and subsequently pulverized using a hammer mill (Retsch HM 200, Haan, Germany) equipped with a 2 mm sieve to ensure a uniform particle size. The extraction was performed according to the procedure established by Bashari *et al*. [8], with specific modifications for optimized phenolic recovery. Briefly, 20 g of the finely ground plant material was treated with 100 mL of 70% aqueous acetone in a 250 mL glass beaker. The mixture was subjected to ultrasonic-assisted extraction for 20 min at 25°C using an ultrasonic water bath (XUB5, Thermo Fisher Scientific, Waltham, MA, USA). The suspension was transferred to centrifuge tubes and centrifuged at 3,000 × *g* for 10 min at 4°C. The supernatant was carefully collected and stored on ice to preserve the stability of the polyphenolic compounds. From 20 g of crude extract was obtained from 20 g of dried raisin pomance (RP) following solvent separation, corresponding to an extraction yield of approximately 10% (w/w, dry basis). To prevent oxidative degradation, all extracts were stored in the dark on ice until analysis was complete.

The total phenolic content of the freshly prepared extract was determined using the Folin-Ciocalteu method described by Makkar *et al*. [9].

### Reagents and standards

- Folin-Ciocalteu reagent (1 N): Commercially available Folin-Ciocalteu reagent (2 N, CAS 12111-13-6) was diluted with an equal volume of distilled water. The solution was stored in a brown bottle at 4°C (gold in color).
- Sodium carbonate (20%): 40 g of sodium carbonate (Na_2_CO_3_·10H_2_O, Sigma-Aldrich, 6132-02-1) was dissolved in distilled water (approximately 150 mL) and made up to 200 mL.
- PVPP: The insoluble polyvinyl polypyrrolidone cross-linked polymer was obtained from Sigma-Aldrich (CAS 25249-54-1).
- Standard tannic acid solution: A stock solution (1 mg/mL) was prepared by dissolving 25 mg of tannic acid (CAS 1401-55-4) in 25 mL of distilled water. A working standard (0.1 mg/mL) was prepared by diluting the stock 1:10 with distilled water immediately before use.

### Preparation of the calibration curve

A standard calibration curve was constructed using increasing tannic acid concentrations. The absorbance was measured at 725 nm.

### Determination of total extractable phenols (TEP)

Aliquots of the RP extract were adjusted to a volume of 0.5 mL with distilled water in test tubes. Folin-Ciocalteu reagent (0.25 mL) and sodium carbonate solution (1.25 mL) were added sequentially. Tubes were vortexed and incubated at 25°C for 40 min. The absorbance was recorded at 725 nm. Total phenols were calculated as **Tannic Acid Equivalents** on a DM basis.

### Determination of non-tannin phenols (TEPH) and tannins

To distinguish simple phenols from tannins, 100 mg of PVPP was weighed into a 100 × 12 mm test tube. Distilled water (1.0 mL) and a tannin-containing extract (1.0 mL) were added. The mixture was vortexed, incubated at 4°C for 15 min, vortexed again, and centrifuged at 3,000 *×* *g* for 10 min. The supernatant containing only non-TEPH was collected. The supernatant’s phenolic content was measured using the Folin-Ciocalteu method described above. A 50 µL (0.050 mL) aliquot of the raisin pomace extract was used in the assay. Absorbance recorded: 0.376. The linear regression equation for these data is as follows:

y = 0.0537x + 0.0027

Where y is the absorbance and x is the total tannin content in µg.

The calibration data are presented in Table 1.

**Table 1:** Calibration curve data used for tannic acid measurements in raisin pomance.

Tube	Tannic acid solution (mL)	Distilled water (mL)	Folin reagent (mL)	Na_2_CO_3_ Solution (mL)	Absorbance (725 nm)	Tannic acid (µg)
Blank	0.00	0.50	0.25	1.25	0.000	0
T1	0.02	0.48	0.25	1.25	0.112	2
T2	0.04	0.46	0.25	1.25	0.218	4
T3	0.06	0.44	0.25	1.25	0.327	6
T4	0.08	0.42	0.25	1.25	0.432	8
T5	0.10	0.40	0.25	1.25	0.538	10

From the standard curve, 0.376 absorbance corresponds to 6.90 µg of TA. Concentration in the extract: 6.90 µg / 0.050 mL = 138 µg/mL = 0.138 mg/mL Total Phenols in Sample: 0.138 mg/mL × 10 mL = 1.38 mg TEP (Total extractable polyphenols) = 1.38 mg/0.2 g sample = 6.90 mg TA/g

### *In vitro* gas production

#### Basal diet nutrient intake, dietary adaptation, and feeding management

The chemical composition of the basal diet (Table 2) was calculated using the manufacturer’s nutrient profiles of the ingredients (Yazd Mokamel Co., Yazd, Iran) and standard output values from the National Research Council (NRC) 2007 standards [10]. Before sample collection, the foals underwent a 14-day adaptation period to a controlled maintenance diet formulated according to the recommendations of the NRC [10]. The ration (basal diet) consisted of a mixture of 1.8 kg of mash-formulated concentrate and 7 kg of lucerne hay (*Medicago sativa*). Meals were provided three times daily at 06:00, 14:00, and 22:00.

**Table 2:** Ingredients and chemical composition of the experimental diets.

Parameter	Treatments^2^			

	C	A	G	A × G
Ingredients and Nutrients^1^				
Chopped lucerne	58.30	58.30	58.30	58.30
Soft straw	25.00	25.00	25.00	25.00
Base concentrate with mineral/vitamin premix	16.70	16.70	16.70	16.70
Chemical composition of treatments^3^				
DM (%)	88.72	88.72	88.72	88.72
CP (%)	12.96	12.96	12.96	12.96
Digestible energy (Mcal/kg)	2.85	2.85	2.85	2.85
NDF (%)	41.48	41.48	41.48	41.48
ADF (%)	33.07	33.07	33.07	33.07
Non-fiber carbohydrates (NFC, %)	22.50	22.50	22.50	22.50
Crude fat (%)	1.70	1.70	1.70	1.70
Ash (%)	7.10	7.10	7.10	7.10
Calcium (%)	1.20	1.20	1.20	1.20
Phosphorus (%)	0.45	0.45	0.45	0.45
Added *Ferula asafoetida* extract equivalent (mg)	0.00	30.00	0.00	30.00
Rassin pomace extract (TA equivalent) (mg)^4^	0.00	0.00	50.00 (0.34)	50.00 (0.34)

1 Commercial vitamin and mineral supplement Active Fit Horse, manufactured by Yazd Mokamel, Iran,

2 Treatments: C (control): basal diet only (no additives); A: basal diet + 5 mL of stock solution A equivalent to 30 mg Asafoetida extract, G: basal diet + 5 mL of stock solution G, equivalent to 50 mg raisin pomace extract, A × G: basal diet + 5 mL of stock solution A + 5 mL of stock solution G,

2 NRC 2007 software estimations,

4 Calculation of TA inclusion in the experimental diet from RP extract. A 5 mL addition (equivalent to 50 mg raisin pomace) provided approximately 0.34 mg TA, assuming 6.90 mg TA g⁻¹ raisin pomace. DM = Dry matter, CP = Crude Protein, ADF = Acid Detergent Fiber, NDF = Neutral Detergent Fiber, NFC = Non-Fiber Carbohydrates, TA = Total tannin.

#### Preparation of the basal diet (substrate)

A basal substrate was prepared from a mixture of 7 kg lucerne hay and 1.8 kg mash-formulated concentrate to simulate the donor diet in the *in vitro* system. Both ingredients were ground separately to pass through a 2-mm mesh screen and subsequently thoroughly mixed by hand in a large plastic vessel to ensure homogeneity. To determine DM content, a 1 kg aliquot of the homogenized basal diet mixture was spread thinly in a pre-weighed Teflon container and dried at 65 °C for 24 hours. The container was reweighed after cooling in a desiccator. Portions of this dried basal diet (300 mg) were weighed into sterile 50 mL serum bottles to serve as the *in vitro* assay substrate.

#### Animal description and justification of age

Four healthy Arabian gelding foals (average age, 14 months; body weight 285 ± 9.65 kg) were selected as donor animals. Before the trial began, horses were checked for parasites and overall health by a certified veterinarian. Growing horses were chosen as the biological model for this study because they possess a developing hindgut microbiome that is particularly susceptible to digestive disturbances and oxidative stress. This heightened sensitivity makes this demographic clinically superior to adult horses for evaluating the efficacy of phytogenic interventions that modulate fermentation kinetics and gut health [11]. The equine hindgut microbiome undergoes significant successional changes and does not reach complete stability until adulthood [12]. Consequently, evaluating phytogenics during this transitional phase is physiologically critical because the developing microbiome is more responsive to dietary modulation and more susceptible to dysbiosis than that of mature horses [13].

The foals were housed in individual, fully ventilated stables equipped with ceiling fans and automated stainless-steel waterers to ensure ad libitum access to fresh water. Stalls were bedded with wood shavings and were cleaned daily. The environmental conditions during the trial (mid-spring 2020) were controlled to maintain a mean ambient temperature of 28°C and a relative humidity of 28%.

#### Manure collection and preparation of cecal microbial inoculum

On day 14 of the adaptation period, fecal samples were collected via rectal stimulation 2 h pre-prandial to minimize post-feeding microbial variations. Approximately 500 g of feces per horse was immediately collected into pre-warmed (39°C), CO_2_-flushed (for 10 min) vacuum flasks to ensure anaerobic conditions. The sealed flasks were transported to the laboratory within 30 min of collection.

Upon arrival, equal-weight aliquots from the four donors were pooled under continuous CO_2_ for 10 mins to create a composite inoculum, a step used to minimize individual host variability. The composite sample was homogenized for 30 s under continuous CO_2_ flushing to maintain anaerobiosis. Subsequently, the homogenate was diluted 1:4 (w/v) with McDougall’s [14] buffer (a bicarbonate-phosphate artificial saliva, pH 6.9), which had been pre-warmed to 39°C and saturated with CO_2_ for 10 mins. The resulting slurry was gently mixed for 1 min and filtered through a double-layered cheesecloth to remove large particulates while retaining the microbial fraction.

#### Inoculation, bottle incubation, and measurement of gas production

The *in vitro* gas production procedure followed the protocol described by Nobari *et al*. [15]. This method utilizes gas accumulation as an indicator of digestion kinetics, reflecting the production of volatile fatty acids (VFAs), CO_2_ methane, and trace hydrogen during anaerobic fermentation. Incubation was initiated by adding the buffered inoculum to the prepared serum bottles (containing substrate and treatments) under anaerobic conditions. Inoculation was performed by dispensing 20 mL of the filtered fecal-buffer mixture into pre-warmed 50 mL serum bottles containing 300 mg of the basal diet as the substrate. This volume results in a headspace of approximately 30 mL per bottle. Six replicates (n = 6) were prepared for each treatment to ensure uniformity and statistical power.

Immediately following inoculation, the headspace was flushed with CO_2_ to establish anaerobic conditions for 60 s, and the bottles were hermetically sealed with butyl rubber stoppers and aluminum crimp caps. The bottles were sealed and maintained at 39°C in an incubator-shaker (Model KM11, Fan Azma Gostar, Iran). Gas volume was measured at 2, 4, 6, 8, 12, 16, 24, 36, 48, 72, 96, and 120 h of incubation using Fedorah and Hrudey’s [16] water displacement method, in which the evolved gas displaces water in graduated tubes connected to the fermentation vessels. To account for non-substrate gas production, six “blank” bottles containing only buffered inoculum were included as negative controls. The net fermentation gas production for each treatment was calculated by subtracting the mean gas volume produced by the blank bottles from the volume recorded for each treatment bottle at each time point.

#### Experimental treatments and stock solutions

The study employed a completely randomized design with a 2 × 2 factorial arrangement, yielding four distinct treatment groups (n = 6 replicates per treatment). Two stock solutions were prepared using distilled water as follows:

1. Stock Solution A (Asafoetida): 6 g of *F. asafoetida* alcoholic extract diluted to 1 L (6 mg/mL; 0.6% w/v).
2. Stock Solution G (tannin): 10 g of grape raisin husk extract diluted to 1 L (10 mg/mL; 1% w/v).

The specific inclusion levels (30 mg for *F. asafoetida* and 50 mg for RPS) were selected based on a preliminary pilot study in our laboratory, which established these concentrations as the optimal range for maximizing fermentation kinetics while maintaining pH stability. The experimental treatments were prepared by adding these solutions to serum bottles containing 300 mg of basal diet as follows:

1. Control (C): A basal diet only (no additives).
2. Treatment A: Basal diet + 5-mL stock solution A equivalent to 30 mg Asafoetida extract
3. Treatment G: Basal diet + 5 mL of stock solution G, equivalent to 50 mg of RP extract
4. Treatment A×G: Basal diet + 5-mL stock solution A + 5-mL stock solution G.

### Statistical analysis

#### Experimental design

The experiment was conducted as a completely randomized design with a 2 × 2 factorial arrangement of treatments. The factors included Asafoetida extract (0 and 30 mg/mL) and RP extract (0 and 50 mg/mL), resulting in four treatment combinations with six replications per treatment (n = 6).

#### Kinetic modeling (nonlinear regression)

Since gas production is a curve (starts slowly, accelerates, and then decelerates), cumulative gas production data recorded over time were fitted to the nonlinear Gompertz equation using the PROC NLIN procedure in SAS 9.4 (SAS Institute, Cary, NC) [17]. The Gompertz equation is the standard biological model for S-shaped growth or fermentation curves. PROC NLIN uses an iterative algorithm (i.e., guessing the parameters, checking the error, adjusting the guess, and repeating) until it finds the “best fit” curve [17]. The Marquardt iterative method was employed to minimize the residual sum of squares and estimate the following kinetic parameters:


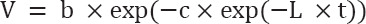


Where:

- V = Cumulative gas production (mL) at time t.
- b = Asymptotic gas production (theoretical maximum, mL).
- c = Specific rate of gas production (mL/h).
- L = Lag phase duration (h).

The goodness of fit for the nonlinear models was assessed by calculating the Pseudo-R² and ensuring convergence of the iteration limit.

#### Parametric analysis

Derived parameters (b, c, L), fermentation pH, NH_3_-N, and digestibility coefficients of nutrients were subjected to analysis of variance using PROC GLM. Before the analysis, the data were screened for normality using the Shapiro-Wilk test (W > 0.90) and for homogeneity of variance using Levene’s test. The statistical model used was as follow:


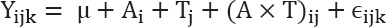


Where:

- Y_ijk_ is the dependent variable.
- μ is the overall mean.
- A_i_ is the effect of Asafoetida.
- T_j_ is the effect of Tannins.
- (A×T)_ij_ is the interaction effect.
- ϵ_ijk_ is the random error.

#### Post-hoc comparisons

In significant F-tests were observed (*p* < 0.05), treatment means were separated using the Tukey–Kramer test to adjust for multiple comparisons and control Type I error. Trends are discussed at 0.05 < *p* < 0.10.

### *In vitro* batch culture

In addition to the gas production trial, an *in vitro* batch culture experiment was conducted using the same basal diet preparation, treatment design, and fecal inoculum protocol described previously in the gas production section. The main distinction of this method was its focus on endpoint measurements of DM, ADF, and NDF disappearance after incubation. For each treatment, 20 mL of the filtered fecal inoculum/buffer mixture was added to 50 mL prewarmed serum bottles containing 300 mg of the basal diet (6 replicates per treatment). After CO_2_ injection to ensure anaerobic conditions for 10 min, the bottles were sealed with rubber stoppers (no foil), fitted with needle tips to release gases, and placed in a shaker incubator at 39°C and 120 rpm. The pH of each bottle was recorded at 2, 4, 6, 8, 12, 16, 24, 36, 48, 72, 96, and 120 h post-incubation. Additionally, eight blank bottles (only buffer and inoculum) were incubated under identical conditions to correct for background DM and fiber loss. After 120 h, all sample and control bottles were removed and centrifuged at 1400 × *g* for 10 min. The supernatant was discarded, and the pellet was washed with a neutral buffer (containing 5.95 g sodium hydrogen phosphate, 0.76 g potassium dihydrogen phosphate, and 2.7 g NaCl in 1 L distilled water) and centrifuged again under the same conditions. The residue was dried at 105 °C, and the DM and crude protein (CP) contents were analyzed. The apparent digestibility of DM (DMD), CP (CPD), ADF (ADFD), NDF (NDFD), or organic matter (OMD) was calculated using the standard formula for *in vitro* nutrient digestibility (IVxD) as described by Nobari *et al*. [15]:


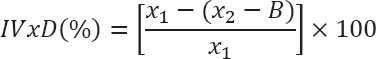


Where:

- *x* represents the specific component being analyzed (DM, CP, ADF, NDF, or OM).
- *x*_1_is the initial weight of the nutrient in the substrate (300 mg basis)
- *x*_2_is the weight of the nutrient in the residue after 120 h incubation.
- *B* is the mean weight of the corresponding nutrient in the blank residues.

#### Blank correction

To determine apparent digestibility, blank correction was performed gravimetrically after drying. The mean residue weight of blank bottles (n = 8 containing only buffered inoculum) was subtracted from the treatment residues. This step corrects for contributing buffer salts and inoculum-derived microbial biomass.

### Quality assurance and experimental replication

To ensure data reproducibility and reliability, strict quality control protocols were implemented throughout the study.

*In vitro* gas production and batch culture experiments were conducted with six analytical replicates per treatment (n = 6). All chemical composition analyses (e.g., phenols, tannins, and GC-MS) were performed in triplicate, and the mean value was reported.

The digital pH meter (Apera PH700-BC, Apera Instruments, USA) was calibrated daily using standard buffer solutions (pH 4.0 and 7.0). Gas volume measurements were validated by ensuring that the water displacement apparatus was leak-proof and equilibrated to atmospheric pressure before readings. The spectrophotometric analysis of tannins relied on a linear standard curve generated using tannic acid (R² = 0.99).

Blank vessels containing only buffered inoculum were included in all runs to correct for non-substrate gas production and residual DM. The bottle placement was randomized within the incubator to eliminate positional bias.

## RESULTS AND DISCUSSION

### Diet composition

As shown in Table 2, all diets had consistent values: DM (88.72%), CP (12.96%), and NDF (41.48%). These are aligned with Brown *et al*. [18], who recommended similar diets with approximately 13% CP and 40% NDF. The forage-to-concentrate ratio of 5:1 (83.33% forage, 16.67% concentrate) complies with the 2007 NRC guidelines for moderate growth, meeting the fiber and energy needs of growing foals, which may require a slight increase for faster growth [19]. NDF and ADF levels (41.48% and 33.07%) are within acceptable ranges. CF (1.7%) is below the recommended 3%–5%, and increasing it is advised for higher energy [20]. The digestible energy (DE) was 2.85 Mcal/kg, which aligns with the NRC 2007 range (2.9–3.2 Mcal/kg). Calcium (1.25%) and phosphorus (0.45%) levels meet NRC 2007 requirements. While the diet adheres to guidelines, a slight increase in protein (13%–14%) and fat (3–5%) is recommended for accelerated growth. Arabian horses are noted for their adaptation to endurance exercise, which may influence their dietary needs, including higher fat utilization compared to other breeds [21]. The CP level of 12.96% meets the NRC 2007 recommendation of 12%–14%.

### Analysis of bioactive compounds

#### R. pomace residues

The chemical composition of risin residues has been analyzed, highlighting their potential as nutrient- and fiber-rich ingredients in animal diets. The findings align with those of prior studies, demonstrating the value of raisin pomace in supporting equine nutrition through its tannin compounds (Table 3). Kolláthov *et al*. [22] reported that raisin pomace supplementation can potentially improve the antioxidant status of the Slovak Warmblood breed.

**Table 3:** Nutritional analysis of raisin pomace residues and the basal diet.

Component	Raisin pomace	Basal Diet
DM (%)	89.28	88.70
CP (%)	10.33	12.90
NDF (%)	31.91	41.40
ADF (%)	25.94	33.00
EE (%)	1.70	2.25
Ash (%)	7.30	7.10
NFC (%)	48.93	22.50
TEP (mg TA/g)	6.90	0.00

*Note: The EE value of 2.25 for the basal diet appears unusually high for a typical lucerne/straw/concentrate ration (crude fat is usually 1–4%). DM = Dry matter, CP = Crude Protein, EE = Ether Extract, ADF = Acid Detergent Fiber, NDF = Neutral Detergent Fiber, OM = Organic Matter, NFC = Non-Fiber Carbohydrates, TEP = Total extractable polyphenols.

#### Asafoetida extract

The chemical profile of Asafoetida comprises approximately 40%–64% resin, 25% gum, and 10%–17% essential oil. The resin fraction is rich in sulfur-containing compounds, such as butyl propenyl disulfide, which contribute to its characteristic aroma and therapeutic efficacy [23]. To the best of our knowledge, this analysis constitutes the first comprehensive GC-MS biochemical profile of *F. asafoetida* specifically established for equine nutritional applications. By identifying key bioactive constituents, such as ϒ-Gurjunenepoxide-(2) (Table 4), specific chemical metabolites can be linked to the observed shifts in hindgut fermentation kinetics.

**Table 4:** Bioactive compounds identified in *Ferula asafoetida* extract.

Category	Compound	Amount (%)
Phenolic Compounds	4-Vinylguaiacol	9.33
	Vanillin	1.44
	Phenol, 2-methoxy-3-(2-propenyl)	0.02
	Vanillic Acid	0.04
Terpenoids	β-Eudesmene	0.07
	γ-Eudesmol	0.08
	α-Bergamotene	0.07
	Elemol	0.03
	Farnesol	0.17
	β-Selinene	0.11
	Caryophyllene	2.17
	Guaiol	0.22
	δ-Cadinene	0.08
Organic and Fatty Acids	Ferulic Acid	3.28
	Linoleic Acid	0.06
	n-Hexadecanoic Acid	0.14
	9-Octadecenoic acid (E)	0.06
Coumarin Derivatives	7-Geranyloxycoumarin	0.25
Biologically active compounds	Digitoxigenin	7.86
	γ-Gurjunenepoxide-(2)	33.68

**Figure 1:**
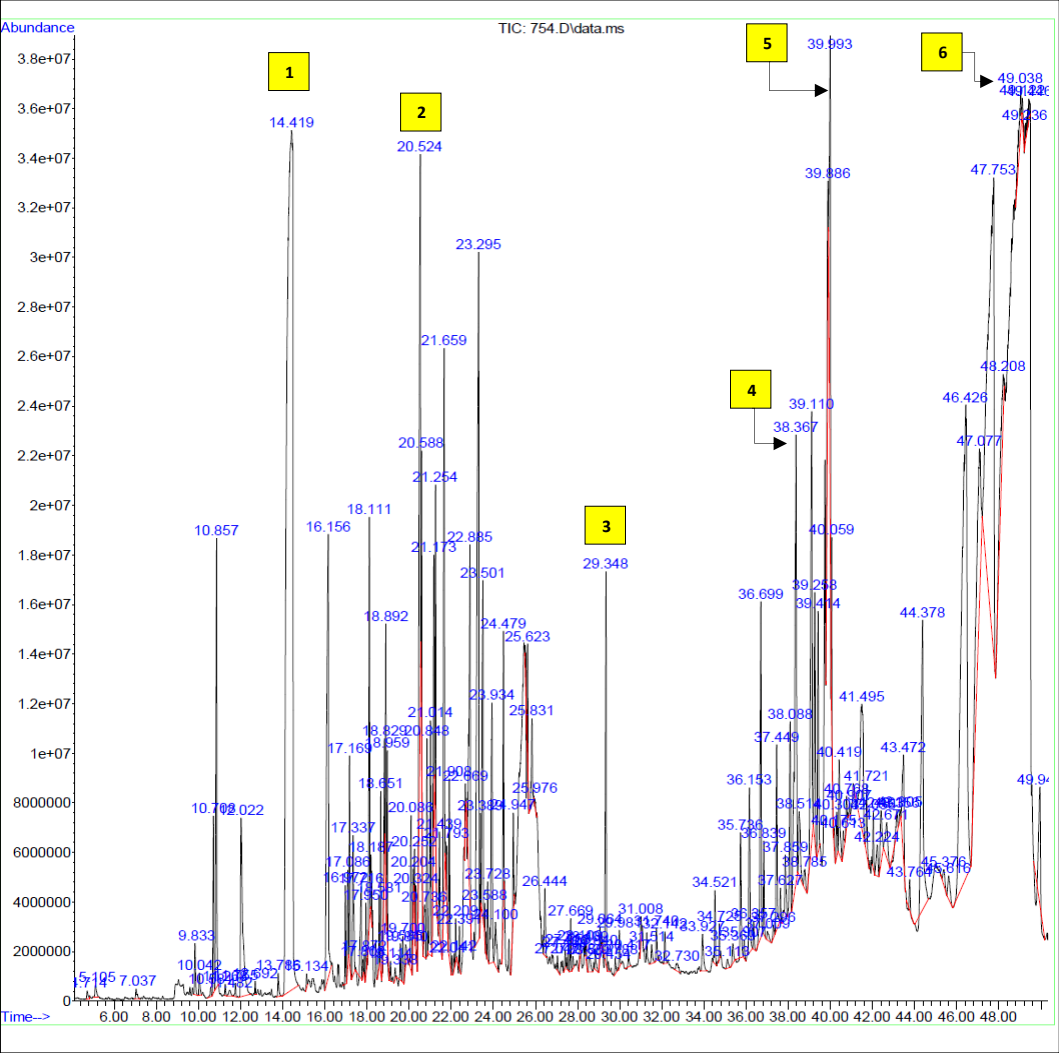
Gas chromatography–mass spectrometry (GC-MS) total ion chromatogram (TIC) of the *F. asafoetida* alcoholic extract. Retention times (RT) are indicated in minutes above the major peaks. Key bioactive compounds, identified based on mass spectral library matching and relative abundance (Table 5), are annotated as follows: (1) 4-Vinylguaiacol (RT: 14.42 min), a phenolic derivative; (2) Caryophyllene (RT: 20.52 min), a bicyclic sesquiterpene; (3) Ferulic acid (RT: 29.35 min), a hydroxycinnamic acid; (4) n-Hexadecanoic acid (RT: 38.37 min), a saturated fatty acid; (5) Digitoxigenin (RT: 39.99 min), a cardenolide; (6) γ-Gurjunenepoxide-(2) (RT: 49.04 min), the primary bioactive sesquiterpenoid (33.68% abundance). The y-axis represents the relative signal abundance.

CO_2_ extracts from the plant’s underground parts are particularly high in unsaturated fatty acids, such as oleic (46.1%) and linoleic acids (43.0%), along with essential amino acids [24]. GC-MS analysis of the *F. asafoetida* extract, based on the NITS mass spectral libraries, revealed a diverse array of bioactive compounds, including coumarins, phenolics, terpenoids, organic acids, and other metabolites with known pharmacological activities (Figure 1, Table 2).

**Table 5:** Effects of the addition of tannin (from raisin pomace) and Asafoetida extract on cumulative gas production.

Treatments^1^	Incubation times (h) and gas production parameters							

	12	24	48	72	120	*b* (mL)	*c* (mL/h*)*	*L* (h)
C	166.00	202.30	220.50	228.80	223.90	217.50	55.40	2.70
A	245.20	284.40	305.60	320.90	321.80	307.10	88.80	3.40
G	286.30	325.90	347.10	355.10	358.50	340.50	150.20	4.00
A × G	211.70	286.40	334.30	347.70	355.60	340.30	187.10	4.50
SEM	1.46	2.06	2.53	2.54	2.66	4.09	0.13	0.00

	**Statistical Effects**							

C vs Others	0.01	0.01	0.01	0.01	0.01	0.01	0.01	0.05
A	0.01	0.01	0.01	0.01	0.01	0.01	0.01	0.01
G	0.01	0.01	0.01	0.01	0.01	0.01	0.05	0.01
A × G	0.01	0.01	0.01	0.01	0.01	0.01	0.01	0.05

1 Experimental Treatments: Control (C): 300 mg dry feed without additive; Treatment A: 300 mg dry feed + 30 mg hydroalcoholic Asafoetida extract; Treatment G: 300 mg dry feed + 50 mg dry raisin pomace; Treatment A × G: 300 mg dry feed + 30 mg hydroalcoholic Asafoetida extract + 50 mg raisin pomace. SEM = Standard error of the mean.

Notably, over 60% of these compounds exhibit anti-inflammatory and antiparasitic properties, underscoring the extract’s potential to modulate gut health (25.92%), reflecting its antioxidant, antimicrobial, and anti-inflammatory properties. Ferulic acid, a prominent constituent, along with polysulfides and coumarins, further supports the pharmacological relevance of the extract [25]. Traditionally used across Iran, Iraq, and Egypt, Asafoetida is used to treat gastrointestinal disorders, including bloating, dyspepsia, and intestinal spasms. Its antispasmodic and antimicrobial effects are well documented [26, 27].

#### Gas production and kinetics of fermentation

The addition of Asafoetida extract (A), condensed tannins (G), and their combination (A × G) significantly influenced the fermentation kinetics (Table 4). The cumulative gas production parameter (V) was highest in treatments G (358.5 mL at 120 h) and A × G (355.6 mL), showing an increase of approximately 53% over the control treatment (228.8 mL), which was significant (*p* < 0.01). The maximum fermentation rate (b) also increased from 55.4 mL in the control to 150.2 mL in G and 187.1 mL in A × G, with corresponding reductions in lag times. The results indicate a marked improvement in gas production and fermentation rates with the addition of *F. asafoetida* extract (A) and condensed tannins (G), either individually or in combination. Treatment G produced the highest cumulative gas volume and maximum fermentation rate, which is consistent with the results of previous studies demonstrating the fermentative efficiency of plant-derived tannins [28]. The A × G combination yielded gas production comparable to that of G alone but showed a higher fermentation rate constant (c).

The observed improvements in fermentation parameters may be attributed to a potential synergistic interaction between the sulfur-containing compounds in Asafoetida and the tannins. Sulfur compounds, such as butyl propenyl disulfide, have been previously documented to possess antimicrobial properties [29]. It is hypothesized that these compounds might modulate the composition of the gut microbiota, potentially creating an environment more conducive to efficient fermentation. However, specific shifts in microbial populations need to be confirmed via genomic analysis. Tannins are widely recognized for their ability to modulate microbial populations, often by selectively inhibiting specific bacterial groups while preserving others [30]. The combination of Asafoetida and tannins may influence digestion through complementary mechanisms [31]. Previous studies on plant-derived bioactive compounds suggest that such combinations can alter the profiles of fermentation by-products, including NH3-N and VFA profiles [32].

The slightly prolonged lag time (L) in the A×G treatment compared to G suggests a period of microbial adaptation to the complex mixture, a phenomenon consistent with observations in other herbivorous species [33].

Although individual VFAs were not quantified by chromatography in this study, GP provides an estimate of aggregate short-chain fatty acid (SCFA) synthesis. In a bicarbonate-buffered system, gas production is stoichiometrically proportional to SCFA generation due to the release of CO_2_ from buffering reactions [34]. The significant increase in the cumulative gas volume observed with the Asafoetida and Tannin treatments suggests a net increase in the overall fermentative activity. Furthermore, the c parameter implies that these additives may have facilitated more rapid fermentation once the initial lag phase was overcome, aligning with the findings of Lowman *et al*. [35] in equine models. We hypothesize that the observed synergy results from a novel dual-action mechanism. The organosulfur compounds in *F. asafoetida* (e.g., disulfides) likely modulate microbial activity through redox interactions, whereas condensed tannins regulate nitrogen availability via reversible protein complexation. This complementary action optimizes the fermentation environment more effectively than either additive alone [36, 37].

#### pH and NH_3_-N

The pH of the fermentation medium remained relatively stable across all treatments during the 120-h incubation period, within the physiological range conducive to fibrolytic activity (Table 6). Although the combined treatment (A × G) recorded the lowest terminal pH (6.33), this value did not fall below the threshold for subclinical acidosis, suggesting that the system’s buffering capacity was not overwhelmed by the accelerated fermentation rates.

**Table 6:** Effects of adding tannin (from raisin pomace) and Asafoetida extract on pH and NH_3_-N.

Treatment^1^	Incubation times (h)								

	2	4	8	12	24	48	72	96	120

	pH Values								
C	6.93	6.80	6.85	6.74	6.61	6.58	6.55	6.42	6.49
A	6.81	6.80	6.74	6.60	6.52	6.54	6.47	6.42	6.32
G	6.85	6.80	6.74	6.63	6.50	6.47	6.42	6.31	6.38
A × G	6.80	6.80	6.76	6.62	6.50	6.42	6.49	6.35	6.35
SEM	0.03	0.02	0.02	0.02	0.02	0.01	0.01	0.01	0.01

	**Statistical Effects**								

C vs Others	0.01	0.01	0.01	0.01	0.05	0.01	0.01	0.01	0.05
A	0.01	0.01	0.01	0.01	0.05	0.01	0.01	0.01	0.05
G	0.01	0.01	0.01	0.01	0.05	0.01	0.01	0.01	0.05
A×G	0.01	0.01	0.01	0.01	0.05	0.01	0.05	0.01	0.05

	**NH_3_-N (mg/dL)**								

C	10.51	12.06	14.30	16.70	18.50	20.16	21.65	23.01	24.58
A	11.05	13.29	15.01	17.40	19.00	21.02	22.45	23.80	25.04
G	11.58	13.85	16.20	18.08	20.50	22.08	23.55	24.80	26.01
A × G	11.34	13.50	16.80	17.80	20.00	21.57	23.30	24.52	25.54
SEM	0.20	0.30	0.35	0.40	0.45	0.50	0.55	0.60	0.65

	**Statistical Effects**								

C vs Others	0.05	0.01	0.05	0.01	0.01	0.01	0.01	0.01	0.01
A	0.01	0.01	0.05	0.01	0.01	0.01	0.01	0.01	0.01
G	0.01	0.01	0.01	0.01	0.01	0.01	0.01	0.01	0.01
A × G	0.01	0.01	0.01	0.01	0.01	0.01	0.01	0.01	0.01

1 Experimental Treatments: Control (C): 300 mg dry feed without additive; Treatment A: 300 mg dry feed + 30 mg hydroalcoholic Asafoetida extract; Treatment G: 300 mg dry feed + 50 mg dry Raisin Pomace; Treatment A × G: 300 mg dry feed + 30 mg hydroalcoholic Asafoetida extract + 50 mg Raisin Pomace.

Regarding nitrogen dynamics, tannin inclusion significantly influenced proteolytic activity. NH_3_-N concentrations were elevated in the G (26.0 mg/dL) and A × G (25.5 mg/dL) treatments compared with the control (24.5 mg/dL; *p* < 0.01). While condensed tannins are often associated with protein-binding that reduces ammonia accumulation, the increased NH3-N levels observed here suggest active microbial protein degradation and turnover. This implies that the specific tannins in RP, at the concentrations used, did not inhibit proteolytic bacteria but instead supported a robust microbial population capable of accessing dietary nitrogen. Alternatively, the sulfur-containing compounds in Asafoetida may have modulated redox potential, further facilitating amino acid deamination [37].

#### Digestibility

Both DMD and CPD were significantly improved in the grape pomace (G) and combined (A × G) treatments compared with the control. This enhancement suggests a favorable modulation of the fermentative environment. Although specific microbial populations were not quantified, previous studies have indicated that tannins can selectively modulate the microbiome, potentially favoring fibrolytic activity and VFA production [38]. The A × G treatment demonstrated comparable benefits, although the values were slightly lower than those with G alone.

The sulfur-rich compounds in *F. asafoetida* may interact with tannins via nucleophilic addition to quinones, possibly moderating their binding capacity or enzymatic inhibition [39, 40]. Consequently, the observed increase in substrate degradation implies a greater potential for VFAs for energy yield. It is important to acknowledge that condensed tannins have a dual nature. While moderate doses are beneficial, high concentrations can exert antinutritional effects, including chelation of essential minerals and formation of irreversible irreversible tannin-protein complexes that reduce nitrogen availability [41]. Therefore, *in vivo* trials are essential to determine the optimal inclusion rates that maximize digestibility without compromising nutrient bioavailability or negatively impacting animal health and performance, while these *in vitro* results indicate improved nutrient accessibility.

In addition, excessive phenolic loads have been linked to reduced feed palatability or even animal health issues in *in vivo* settings [42]. However, the significant improvement in CPD in the current study suggests that the 50 mg inclusion level remained below the threshold for these harmful effects, likely modulating proteolysis without compromising nutrient accessibility.

### Limitations

The absence of 16S rRNA sequencing to profile specific taxonomic shifts is a primary limitation of this study. However, gas production kinetics provide a valuable readout of the functional capacity of the microbiome. The observed significant reduction in the gas production L parameter and increased c parameter indicates accelerated microbial colonization and enzymatic hydrolysis [43]. This aligns with the concept of functional redundancy, in which metabolic output (such as enhanced digestibility) is optimized by additives even when specific bacterial populations shift or remain uncharacterized [44].

However, the inherent constraints of the *in vitro* model must be acknowledged. The static batch culture represents a closed environment that lacks the continuous digesta flow and mucosal absorption mechanisms of the living equine hindgut. Consequently, the accumulation of fermentation end-products may differ from the physiological conditions where VFAs are rapidly absorbed. In addition, the short-term incubation period (96 h) precludes the assessment of potential long-term microbial adaptation or resistance to the bioactive compounds. Finally, although dosage concentrations were selected based on safety thresholds reported in previous literature, this study did not perform direct cytotoxicity assays on equine intestinal epithelial cells to verify tissue safety limits experimentally.

## CONCLUSION

The hydroalcoholic extract of *F. asafoetida* and condensed tannins from raisin pomace significantly enhanced *in vitro* fermentation kinetics in equine hindgut models, with cumulative gas production increasing by up to 53% in tannin-supplemented treatments compared to controls (*p* < 0.01). Key bioactive compounds identified via GC-MS, such as γ-Gurjunenepoxide-(2) (33.68%) and ferulic acid (3.28%), contributed to improved DMD and CPD. The pH remained stable (6.3–6.9), while NH_3_-N levels rose moderately (24.5–26.0 mg/dL), indicating active microbial protein turnover without acidosis risk. Synergistic effects in the combined treatment (A × G) yielded the highest fermentation rate (187.1 mL/h), underscoring the potential of these phytogenics to optimize nutrient utilization.

These findings suggest that *F. asafoetida* extract and raisin pomace tannins could serve as natural feed additives to improve hindgut fermentation and nutrient digestibility in growing horses, potentially reducing reliance on synthetic supplements and enhancing gut health in equine nutrition. At inclusion levels of 30 mg and 50 mg, respectively, they offer a cost-effective means to boost energy yield from forages, particularly for Arabian foals prone to oxidative stress. However, translation to practical feeding requires validation in *in vivo* settings to ensure palatability, mineral bioavailability, and long-term performance benefits, aligning with sustainable agriculture practices.

A major strength of this study is its pioneering GC-MS profiling of *F. asafoetida* for equine applications, providing a detailed chemical basis for observed effects. The 2 × 2 factorial design robustly demonstrated synergistic interactions, supported by replicated *in vitro* assays that closely mimic hindgut conditions. Alignment with NRC guidelines and use of fecal inoculum from target animals enhance translational relevance.

The *in vitro* model limits extrapolation to dynamic *in vivo* systems, lacking continuous digesta flow and absorption. Absence of 16S rRNA sequencing hinders microbial insights, while short incubation periods (120 h) overlook long-term adaptation. No direct cytotoxicity testing or VFA quantification further constrains comprehensive safety and mechanistic evaluation.

Future research should conduct *in vivo* trials in growing horses to assess optimal dosing, palatability, and impacts on growth performance and microbiome diversity via metagenomics. Exploring dose-response curves, interactions with varied diets, and long-term health effects could refine applications. Comparative studies across breeds and integration with other phytogenics may broaden scope.

In summary, *F. asafoetida* extract and raisin pomace tannins exhibit promising potential as phytogenic additives for enhancing equine hindgut fermentation and digestibility. While *in vitro* results highlight synergistic benefits, *in vivo* validation is crucial to harness their full practical value in sustainable equine nutrition.

## DATA AVAILABILITY

All the generated data are included in the manuscript.

## AUTHORS’ CONTRIBUTIONS

BN: Conceptualized the study. SMA: Developed the experimental design. HD: Performed wet-chemistry analyses, including the preparation of *F. asafoetida* and raisin pomace extracts, collection of equine fecal inoculum, and daily monitoring of the *in vitro* gas production system. BN and SMA: Performed data curation and statistical analysis. PS and HHF: Interpreted the results of GC-MS and digestibility. All authors have participated in drafting and revision and approved the final manuscript.
